# Shaping postoperative outcomes: microbiota-modifying dietary fiber interventions in colorectal cancer treatment

**DOI:** 10.3389/fmicb.2026.1774501

**Published:** 2026-06-24

**Authors:** Claire McCartney, Roy Hajjar, Manuela M. Santos

**Affiliations:** 1 Nutrition and Microbiome Laboratory, Institut du Cancer de Montréal, Centre de Recherche du Centre Hospitalier de l’Université de Montréal (CRCHUM), Montréal, QC, Canada; 2 Digestive Surgery Service, Centre Hospitalier de l’Université de Montréal, Montréal, QC, Canada; 3 Department of Surgery, Université de Montréal, Montréal, QC, Canada; 4 Department of Medicine, Université de Montréal, Montréal, QC, Canada

**Keywords:** colorectal cancer, colorectal surgery, dietary fiber, gut barrier, gut microbiota, inflammation, prebiotics

## Abstract

The gut microbiota plays a key role in intestinal homeostasis by reinforcing the gut barrier and modulating inflammation. Gut barrier dysfunction or dysregulated inflammatory response in patients with colorectal cancer may lead to an increased risk of surgical complications associated with poor intestinal healing. Microbiota-modifying dietary interventions have the ability to shift microbial community structures and bacterial metabolite production, with profound implications for host health. This review focuses on the potential for short-term, preoperative, dietary fiber interventions in improving colorectal cancer surgical and oncological outcomes. Additionally, this review highlights important considerations for the optimization and personalization of dietary fiber interventions, notably individual- and fiber-specific microbiota responses.

## 1 Introduction

Beginning in the 1970s, widespread preventative screening programs aimed at the “average-risk” section of the population between 50 and 74 years of age have contributed significantly to the decline in colorectal cancer (CRC) incidence and death rates (Edwards et al., 2010). The use of fecal occult blood tests for early detection of CRC gained popularity in the 1980s, followed by the wide use of colonoscopies in the 1990s that could detect both cancer and pre-cancerous polyps (Edwards et al., 2010). Given that CRC prognosis is strongly correlated with the stage at diagnosis, screening has also led to a decrease in CRC mortality, as the disease is often discovered at an earlier stage. Increasing awareness of risk factors, such as smoking and physical inactivity, and treatment optimization have also contributed to this decline. Over the past decade, CRC has seen the largest decline in new cases compared to previous years, with incidence rates decreasing by 4% each year in men and by 3.1% in women since 2014 (Canadian Cancer Statistics, 2023).

Despite declining rates in the older population, CRC is still the third most prevalent cancer worldwide as of 2022, with an age standardized rate (ASR) of 10.7 new cases per 100,000 per year, as well as the second leading cause of cancer related death, with an ASR of 4.7 (Ferlay et al., 2024). In addition, this overall trend disguises an alarming increase in CRC cases among younger individuals, mainly seen in Canada, the US, and other countries with a high socioeconomic index (Brenner et al., 2019; Araghi et al., 2019; Sung et al., 2025). Early-onset CRC (EO-CRC) is generally defined as CRC occurring in individuals less than 50 years of age. While cases of EO-CRC make up a small percentage of total CRC cases, the incidence of EO-CRC has continued to increase between 2 to 4% each year since the 1990s. Colon cancer diagnoses among younger individuals have increased 40%, and rectal cancer diagnoses have increased an alarming 90% during the same time period (Stoffel and Murphy, 2020). Conservatively, it is estimated that by 2030 11% of colon cancer cases and 23% of rectal cancer cases will occur in those younger than 50 years (Bailey et al., 2015). As of yet, this increase in EO-CRC remains unexplained.

Ultimately, CRC is expected to remain a serious global health concern in the coming decades and the optimization of current treatment strategies is required to meet this need. Crucially, alterations in the gut microbiota (Bautista et al., 2026) and changes in dietary habits, notably a lack of dietary fiber intake (Wu et al., 2016), have been implicated in CRC development, but their potential role in CRC treatment has been less explored.

## 2 Colorectal cancer etiology and risk factors

The vast majority (>95%) of CRCs begin as abnormal premalignant lesions in the colon or rectum, also termed “adenomas” or “polyps” (Shussman and Wexner, 2014). While initially benign, accumulating mutations drive clonal expansion and adenoma progression through intermediate and late stages, before developing into carcinomas (Schmitt and Greten, 2021). Many factors contribute to CRC development, which is believed to occur over a long period of time – for most patients, the cancer found at diagnosis will have probably first appeared 5–15 years prior (Sawicki et al., 2021).

Multiple factors may contribute to CRC development, including modifiable and non-modifiable risk factors. Important modifiable risk factors include several lifestyle-related contributors, such as sedentary behavior, obesity, smoking, and high alcohol consumption, that have consistently been associated with increased risk (Chu et al., 2025). Non-modifiable risk factors include hereditary genetic syndromes such as Lynch syndrome (Abu-Ghazaleh et al., 2022) and familial adenomatous polyposis (Stoffel and Murphy, 2020), as well as somatic mutations that accumulate with age, making advancing age itself a major determinant of CRC incidence.

Chronic inflammation is another well-established driver of cancer development, highlighted by the fact that patients with inflammatory bowel disease (IBD) are two to three times more likely to develop CRC (Jess et al., 2005). Revealingly, the risk of developing colitis-associated CRC (CAC) increases with the severity and extent of the inflammation, as well as the number of years lived with the disease (Stidham and Higgins, 2018). During inflammation, immune cells release reactive oxygen species (ROS) and reactive nitrogen species (RNS) that can induce genome-wide DNA damage in intestinal epithelial cells (Meira et al., 2008). By affecting key genes implicated in adenoma-to-carcinoma pathogenesis (Shah and Itzkowitz, 2022), mutations caused by chronic inflammation are sufficient for tumorigenesis, even in the absence of exogenous carcinogenic substances (Canli et al., 2017). Aside from IBD, other sources of chronic inflammation include infection, dysfunctional immune reactions and environmental triggers, such as airborne pollutants, smoking, and poor diet (Grivennikov et al., 2010). Although chronic inflammation is a clear contributing factor in only 5% of CRC cases, between 70 and 90% of sporadic cases can be attributed to various pro-inflammatory environmental factors, notably “Western-style” diets high in processed meats, fat and refined sugar, and low in fiber (Wu et al., 2016).

Dietary habits are among the most influential factors shaping the composition and function of the gut microbiota, a complex community of bacteria, archaea, fungi, and viruses that reside primarily in the human colon (Bull and Plummer, 2014). The gut microbiota plays an essential role in host physiology by contributing to nutrient metabolism (Oliphant and Allen-Vercoe, 2019), immune system modulation (Wu and Wu, 2012), and maintenance of intestinal barrier integrity (Di Vincenzo et al., 2024). Among dietary components, fibers represent a key modulator of microbial ecology (Rinninella et al., 2023), as many fiber types escape digestion in the upper gastrointestinal tract and serve as substrates for microbial fermentation in the colon.

## 3 The gut microbiota in host health and disease

The human gut microbiota is made up of more than 10^13^ microorganisms (Sender et al., 2016), together encoding more than 150x the genetic material found in the human genome (Cantarel et al., 2012). It is therefore no surprise that the gut microbiota plays a significant role in both human health and disease. Described by many as a “virtual organ” (Shanahan, 2002; Evans et al., 2013; O'Hara and Shanahan, 2006), the gut microbiota plays an active role in immune development and regulation (Zheng et al., 2020), energy homeostasis (Corbin et al., 2023) and neuroendocrine function (Evans et al., 2013). The major phyla present in the human gut include Bacteroidetes and Firmicutes, accounting for roughly 90% of bacteria, as well as Actinobacteria and Proteobacteria, which make up the remaining 10% (Arumugam et al., 2011). Many factors, such as host genetics as well as diet, antibiotic use, and other environmental factors shape the relative composition of the gut microbiota, which is highly personalized and has been shown to vary considerably between individuals (Mills et al., 2019a). This profound connection between the host and its associated microbial communities has been exemplified by the inclusion of the microbiome, with particular emphasis on the gut microbiome, as one of the hallmarks of cancer (Hanahan, 2022).

### 3.1 Pro-carcinogenic bacteria

Efforts to characterize the human gut microbiome over the past several decades have identified several bacteria species that are more abundant in CRC patients, including *pks+ Escherichia coli*, enterotoxigenic *Bacteroides fragilis*, and *Fusobacterium nucleatum* (Bautista et al., 2026), while other beneficial taxa, including the butyrate producing *Faecalibacterium prausnitzii*, *Roseburia*, *Lactobacillus* and *Lachnospiraceae* species, were found to be less abundant (Thakur et al., 2026). While it remains difficult to establish a direct causal role for many taxa in CRC development, certain species produce toxins and other virulence factors that, while intended to help compete against other bacteria, also have the ability to induce DNA mutations in host cell CRC-driver genes, activate proliferation pathways (Rubinstein et al., 2013), or promote a pro-tumorigenic immune microenvironment (Chung et al., 2018).

#### 3.1.1 *pks* + *Escherichia coli*

*E. coli* is a common commensal bacterium of the digestive tract. However, certain *E. coli* strains within the B2 phylogenic group harbor a gene cluster termed polyketide synthase or ‘*pks*’ that encodes the enzymes necessary for the production of colibactin, a secondary metabolite (Nougayrède et al., 2006; Dougherty and Jobin, 2021). Colibactin translocates to the nucleus of colon epithelial cells, forming DNA adducts and leading to the formation of inter-strand crosslinks in adenine-rich DNA regions as well as DNA double-strand breaks (DSB) (Xue et al., 2019). Attempts to repair these damages result in a specific mutational signature characterized by an increase in T > N single-base substitutions (SBS) within AT-rich regions as well as short insertion/deletions (Dziubańska-Kusibab et al., 2020; Pleguezuelos-Manzano et al., 2020). In Caco-2 human adenocarcinoma cells exposed to *pks+ E. coli*, these mutational signatures were most likely to be observed in the *APC* tumor suppressor gene commonly mutated in CRC (Pleguezuelos-Manzano et al., 2020). Mutations in key signaling pathways, including p53 and Wnt, along with enhanced cell proliferation and impaired differentiation, were also observed in human colon organoids exposed to *pks*+ *E. coli* (Iftekhar et al., 2021). In pre-clinical CAC models, *pks+ E. coli* colonization increased tumor initiation (Yang et al., 2020) and tumor progression (Arthur et al., 2012), as well as the expression of the phosphorylated histone protein gH2AX, a marker for DNA DSB.

#### 3.1.2 Enterotoxigenic *Bacteroides fragilis*

In contrast to the human commensal non-toxigenic *Bacteroides fragilis*, which demonstrates anti-inflammatory properties and promotes immunological tolerance (Round et al., 2011), enterotoxigenic *Bacteroides fragilis* (ETBF) is commonly associated with both inflammatory bowel disease (Prindiville et al., 2000) and CRC development (Cao et al., 2021). ETBF produces the metalloprotease *B. fragilis* toxin (*bft*) that binds to intestinal epithelial cells and rapidly degrades E-cadherin, leading to the activation of Wnt/b-Catenin signaling pathways and persistent cell proliferation (Wu et al., 2003). Other carcinogenic mechanisms of *bft* include the activation of signal transducer and activator of transcription 3 (STAT3) and recruitment of T helper type 17 (T_h_17) cells, leading to increased inflammation, colitis, and tumor formation (Wu et al., 2009). CRC patients, especially those with later stage cancer, show higher levels of fecal (Haghi et al., 2019) and mucosal (Boleij et al., 2014) ETBF. Notably, ETBF has been measured at higher levels in fecal samples from patients with early stage adenomas, demonstrating its use as a potential biomarker for early detection of CRC (Périchon et al., 2022).

#### 3.1.3 Fusobacterium nucleatum

*Fusobacterium* species, which make up <0.01% of the gut microbiota composition in healthy individuals (Segata et al., 2012), are more abundant in stool and enriched in tissue samples from patients with adenocarcinomas (Kostic et al., 2013). *F. nucleatum* promotes CRC development by directly binding to and inhibiting E-cadherin using the Fusobacterium A (FadA) adhesin, upregulating *β*-catenin signaling pathways involved in proliferation and pro-inflammatory cytokine production (Rubinstein et al., 2013). *F. nucleatum* was also shown to modulate the tumor microenvironment (TME) through the recruitment of immunosuppressive myeloid-derived immune cells and pro-inflammatory M2 tumor-associated macrophages (TAM) (Kostic et al., 2013). Additionally, *F. nucleatum* was shown to promote a T_h_17-mediated pro-tumorigenic response similarly to ETBF (Brennan et al., 2021). Finally, *F. nucleatum* may play a role in CRC metastasis by upregulating CCL20-mediated cancer cell migration, infiltration and TME remodeling (Guo et al., 2021), with tumor *F. nucleatum* abundance being correlated with worse patient clinical outcome, highlighting its potential as a prognostic biomarker (Mima et al., 2016).

### 3.2 Gut barrier reinforcement

The gut microbiota plays a significant role in maintaining the integrity of the intestinal barrier, a highly regulated interface between the external environment, which is contiguous with the GI tract, and the body’s internal environment. The outmost section of the gut barrier consists of a protective mucus layer secreted by goblet cells (Corfield, 2015). The physical gut barrier is provided by a layer of intestinal epithelial cells connected via adherens junctions, gap junctions, desmosomes and tight junctions (Groschwitz and Hogan, 2009). Tight junctions, which include occludins, claudins, and zonula occludens, are the membrane proteins primarily responsible for the regulation of intestinal selective permeability, which allows for the uptake of nutrients, water and electrolytes, to the exclusion of pathogens, toxins, and foreign antigens (Kunzelmann and Mall, 2002). Highly specialized Paneth cells secrete antimicrobial compounds upon recognition of pathogen-associated molecular patterns (PAMPs), such as the bacterial outer membrane and cell wall components lipopolysaccharide (LPS) and peptidoglycan (Akira et al., 2006). Finally, there exists an immunological barrier comprised of innate and adaptive immune cells within the lamina propria underlying the epithelium (Mowat and Agace, 2014). Overall, the gut barrier is a complex system that balances immune system education and protection against pathogens.

### 3.3 Bacterial metabolites

Beyond a direct influence on CRC initiation and progression, the gut microbiota exerts many of its effects through the generation of metabolites. Microbial fermentation of dietary substrates, particularly fibers, yields short-chain fatty acids (SCFA), which serve as key energy sources for colonocytes, reinforce epithelial integrity, and modulate immune responses. In addition, gut microbes are also deeply involved in bile acid transformation, converting primary bile acids into secondary bile acids with distinct signaling properties that influence metabolic, inflammatory, and barrier functions.

#### 3.3.1 Short-chain fatty acid production

SCFA are carboxylic acids with aliphatic tails containing up to 6 carbons produced mainly by the bacterial fermentation of dietary fibers in the colon (Layden et al., 2013). The vast majority (~95%) of SCFA are absorbed in the colon, with the total concentration of SCFA being at its highest in the cecum and proximal colon and decreasing toward the distal colon (Cummings et al., 1987). The most abundant SCFA are acetate (two-carbon backbone – C2), propionate (three-carbon backbone – C3) and butyrate (four-carbon backbone – C4), present in a ratio of roughly 60:20:20, respectively (Cummings et al., 1987). Following the breakdown of complex carbohydrates by gut bacteria, the resulting monosaccharides may enter one of multiple SCFA biosynthesis pathways (Koh et al., 2016). Acetate is produced from pyruvate via acetyl CoA, while butyrate is synthesized from two molecules of acetyl CoA (Beisner et al., 2021). Propionate is mainly produced via the succinate pathway but can also be produced via the acrylate pathway from lactate, or via the propanediol pathway, which makes use of fucose and rhamnose sugars (Louis et al., 2014). Additionally, in the absence of dietary fiber, gut bacteria may ferment diet- or host-derived amino acids, leading to the production of branched-chain SCFA, including isobutyrate, 2-methylbutyrate, and isovalerate (Smith and Macfarlane, 1997).

SCFA activate the G-protein-coupled receptors (GPCR) GPR41 (free fatty acid receptor 3; FFAR3), GPR43 (free fatty acid receptor 2; FFAR2) and GPR109A which are expressed to varying degrees on a variety of cell types, including colon epithelial cells and adipocytes, as well as cells of the innate and adaptive immune system (Mann et al., 2024). In addition, these GPCR differ in their affinity for each SCFA, with GPR41, widely expressed in multiple tissues, preferentially binding propionate, GPR43, primarily found in immune cells, binding acetate, propionate and butyrate, and GPR109A, largely expressed in the colon, predominantly activated by butyrate (Mann et al., 2024). These differences in tissue expression and affinity help explain the diverse homeostatic functions of SCFA, as evidenced by their dysfunction in several disease states, including obesity and other metabolic disorders, CRC and IBD (Koh et al., 2016; Machiels et al., 2014). In addition to GPCR binding, SCFA may directly enter cells through passive diffusion or by way of the transmembrane transporters monocarboxylate transporter 1 (MCT1, encoded by *SLC16A1*) or sodium-coupled monocarboxylate transporter 1 (SMCT1, encoded by *SLC5A8*) (Sivaprakasam et al., 2017). Direct entry of SCFA into cells allows for butyrate and, to a lesser extent, propionate, to regulate transcription through epigenetic modifications, notably through the inhibition of histone deacetylases (HDAC) (Thomas and Denu, 2021).

SCFA serve as a key link between the gut microbiota and many important physiological functions, including host metabolism, gut barrier integrity, intestinal immune activity and cell proliferation (Koh et al., 2016; Mann et al., 2024). Butyrate and acetate were shown to promote intestinal homeostasis by inducing antimicrobial peptide (AMP) production through GPCR43 activation of mTOR and STAT3 pathways (Zhao et al., 2018). SCFA were also shown to increase colon epithelium oxygen consumption and activate hypoxia-inducible factor (HIF) regulated pathways involved in mucin production, tight junction protein expression and pathogen clearance (Kelly et al., 2015). The anti-inflammatory activity of SCFA is exemplified by GPR109A activation on colon epithelial cells and colon resident macrophages resulting in the production of IL-18 and IL-10 and subsequent regulatory T cell (Treg) differentiation and reduction of inflammation (Singh et al., 2014). Both butyrate and propionate were shown to increase Treg expansion and differentiation through both GPCR- and HDAC-mediated activity (Arpaia et al., 2013). Butyrate was also shown to decrease the production of pro-inflammatory nitric oxide (NO), IL-6, and IL-12 by macrophages in the colon lamina propria (Chang et al., 2014).

Compelling evidence has been published in recent years regarding the beneficial role of SCFA in CRC. Butyrate specifically has been shown to promote both anti-inflammatory and anti-carcinogenic effects through activation of the nuclear receptor peroxisome proliferator-activated receptor gamma (PPAR-*γ*) (Hajjar et al., 2024c). Through its function as an HDAC inhibitor, butyrate is able to paradoxically stimulate the proliferation of healthy colorectal epithelial cells while inhibiting that of cancerous cells via epigenetic promotion of tumor suppressors and inhibition of proto-oncogenes (Hajjar et al., 2021b, 2024c). The beneficial effects of butyrate in CRC surgical treatment and prevention of cancer recurrence and metastasis were shown in the context of fiber supplementation in preclinical murine models of colorectal surgery and liver metastasis (Hajjar et al., 2024b).

#### 3.3.2 Bile acid transformation

Primary bile acids are cholesterol-derived molecules synthesized in the liver, consisting of cholic acid (CA) and chenodeoxycholic acid (CDCA) (Sayin et al., 2013). Primary bile acids are further conjugated in the liver with glycine or taurine, allowing them to become fully ionized at intestinal pH (Ridlon et al., 2016). Conjugated bile acids are stored in the gallbladder as a component of bile and released into the duodenum after food consumption. In the small intestine, ionized bile acids, termed bile salts, act as detergents to emulsify dietary fats, facilitating their absorption (Ridlon et al., 2016). Roughly 95% of bile acids are reabsorbed in the small intestine, most as bile salts in the terminal ileum via the high-affinity apical sodium dependent bile acid transporter (ASBT), after which they are transported via the portal vein to the liver and recycled (Wahlström et al., 2016). The small fraction of bile acids that reach the colon are deconjugated by gut bacteria via bile salt hydrolases (BSH), inhibiting their re-uptake (Ridlon et al., 2006). BSH are highly conserved enzymes among gut bacteria and are present across all major phyla.

Deconjugated bile acids are transformed via 7α-dehydroxylation into secondary bile acids, a metabolic capability that is conferred by the bile acid-inducible (*bai*) bacterial operon (Funabashi et al., 2020). The secondary bile acid deoxycholic acid (DCA) is derived from CA, while lithocholic acid (LCA) and ursodeoxycholic acid (UDCA) are derived CDCA (Eyssen et al., 1983; MacDonald et al., 1983). More recently, in addition to deconjugated bile acids and secondary bile acids, a new class of microbially conjugated bile acids (MCBA) was identified and shown to be increased in fecal samples of patients with IBD (Quinn et al., 2020).

Bile acid levels are tightly regulated via a negative feedback loop initiated by CA, CDA, LCA or DCA binding to the farsenoid X nuclear receptor (FXR) which is expressed in several tissues, including the liver and intestine (Makishima et al., 1999). Activation of FXR leads to the secretion of fibroblast growth factor 19 (FGF19) in the portal vein, where it travels to the liver and inhibits bile acid synthesis via the activation of FGF receptor-4 (Inagaki et al., 2005). FXR, along with the additional bile acid receptors Takeda G-protein coupled receptor 5 (TGF5), vitamin D receptor (VDR), pregnane X receptor (PXR) and constitutive androstane receptor (CAR) have been shown to play important immunomodulatory roles (Thibaut and Bindels, 2022). Notably, FXR (Vavassori et al., 2009), PXR (Shah et al., 2007), and TGF5 (Wang et al., 2011) activation has been shown to suppress NF-κB mediated inflammation, while activation of the FXR also reduced IL-6 and TNF-*α* secretion and decreased intestinal permeability in a DSS-induced colitis model (Gadaleta et al., 2011).

Due to their primary function as detergents, bile acids are hydrophobic in nature and therefore damaging to colon epithelial cells (Hofmann, 1999), potentially contributing to CRC development via epithelial barrier damage and promotion of chronic inflammation (Shi et al., 2023). The hydrophobicity and toxicity of bile acids depend on their structure, with LCA and DCA being the most and UDCA the least hydrophobic (Hofmann, 1999). UDCA was shown to have a cytoprotective role and has been associated with a lower rate of neoplasia in patients with UC (Tung et al., 2001). In contrast, DCA has been detected at elevated levels in the feces and serum of patients with CRC (Bernstein et al., 2005) and has more recently been implicated in the development of obesity-associated liver cancer via the induction of a pro-inflammatory senescence associated secretory phenotype (SASP) (Yoshimoto et al., 2013).

Finally, the diet, in addition to directly modifying bile acid metabolism, also modifies the gut microbiota composition and enzymatic capacity, indirectly affecting the composition of the bile acid pool. The consumption of cholesterol rich foods increases the synthesis and secretion of primary bile acids, leading to the accumulation of secondary bile acids in the colon, as well as increased oxidative stress and inflammation (Stadler et al., 1988). High-viscosity dietary fibers including *β*-glucan have also been shown to sequester cholesterol and bile acids in the colon, leading to increased bile acid synthesis and excretion (Ellegård et al., 2007), however the effect of this sequestration on secondary bile acid-associated epithelial damage and its impact on colorectal surgical recovery has yet to be explored.

## 4 Microbiota-modifying dietary fibers

Given the numerous ways in which the gut microbiota impacts host physiological functions, both locally in the colon and systemically, efforts to shape the gut microbial community have emerged as attractive options for the mitigation of various health issues. Chief among them is the use of prebiotics, which are described as “substrate[s] that [are] selectively utilized by host microorganisms conferring a health benefit’ (Gibson et al., 2017). Prebiotics are unable to be metabolized by host enzymes and remain undigested and unabsorbed in the small intestine until reaching the colon, where they are fermented by gut bacteria. Most prebiotics are carbohydrate-based, however other compounds, such as the polyphenol curcumin, have also been identified (Hassaninasab et al., 2011).

Dietary fibers are carbohydrates that are neither digested nor absorbed in the small intestine which exert a physiological benefit (Jones, 2013). Dietary fibers may be naturally occurring in foods, processed physically, chemically or enzymatically, or synthetically produced (Jones, 2013). There is no consensus as to the required structural complexity of dietary fiber, with some countries, including Canada, instead defining a dietary fiber as a nondigestible carbohydrate containing at least 3 monomers (Jones, 2013). Dietary fibers can be classified according to their origin, physical structure, and physicochemical properties, including their solubility, viscosity and fermentability, which in turn impact their physiological functions (Ariyarathna et al., 2025). While most prebiotics are dietary fibers, not all dietary fibers can be classified as prebiotics (Slavin, 2013). In order for a dietary fiber to be considered a prebiotic, the health benefit conferred by the fiber must be due in part to the selective modulation of the gut microbiota.

### 4.1 Bacterial CAZymes

The fermentation of dietary fiber by gut bacteria is a prime example of the symbiotic relationship between the gut microbiota and the host. The human genome contains a limited number of glycosidases capable of breaking down complex carbohydrates, with degradative activity limited to starches, sucrose and lactose via amylase, sucrase and lactase, respectively (Martens et al., 2011). Undigested dietary fibers are fermented by gut bacteria in the colon, leading to the formation of metabolic end products such as SCFA that can be utilized by the host (Flint et al., 2012b).

The gut microbiome comprises 150 times more genes than the human genome, including a wide variety encoding carbohydrate degradative enzymes (Cantarel et al., 2012). A number of bacterial carbohydrate active enzymes (CAZymes) have been and continue to be identified using functional metagenomic sequencing (Tasse et al., 2010). CAZymes vary considerably across phyla down to the individual strain (Flint et al., 2012b). The most prevalent types of CAZymes are glycoside hydrolases (GH), followed by glycoside transferases (GT), polysaccharide lyases (PL) and carbohydrate esterases (CE), as well as carbohydrate binding molecules (CBM) that facilitate CAZyme to substrate binding (Cantarel et al., 2009). For example, inulin degradation is carried out by inulase (GH family 32) or inulin lyase (GH91), which cleave *β*-(2-1) linked fructose molecules, as well as β-fructofuranosidase (GH32) which cleaves the terminal *α*-(1-2) linkage of fructose and glucose (Flint et al., 2012b). A collection of 8 genes that make up the starch utilization system (Sus) in *Bacteroides thetaiotaomicron* was the first polysaccharide utilization locus (PUL) to be characterized (Anderson and Salyers, 1989). The Sus model of degradation involved the initial extracellular cleavage of starch into large oligosaccharides, which were transported across the cell membrane before being broken down into monosaccharides (Anderson and Salyers, 1989). This model of degradation has been described as ‘selfish’ and prevents other bacteria from accessing the end products of degradation (Cuskin et al., 2015). Most bacteria capable of breaking down complex carbohydrates are found within the Actinobacteria and Firmicutes phyla (Deehan et al., 2017). However, extensive cross-feeding networks are also common among gut microbiota, in which certain bacteria (keystone species) initiate carbohydrate breakdown while other bacteria, which lack the CAZymes required for initial cleavage, complete the degradative process (Rakoff-Nahoum et al., 2014).

The structural complexity of a complex carbohydrate dictates which CAZymes are needed for it to be broken down into its respective monosaccharides, and by extension which bacteria are required to be present within the microbial community. For example, the degradation of rhamnogalacturonan II, a component of pectin, requires 21 distinct CAZymes, and 3 different PUL (Ndeh et al., 2017). Utilization of this polysaccharide is generally achieved via cross-feeding as only certain bacteria, such as *B. thetaiotaomicron*, carry most of the required CAZymes. In the context of surgical healing, the presence of certain “keystone” species required for the degradation of more complex fibers and their use by other SCFA-producing taxa may serve as potential biomarkers that could predict patient response to a dietary fiber intervention aiming specifically to increase SCFA concentrations and improve gut barrier integrity prior to surgery.

### 4.2 Fiber specificity

The ability to generate a consistent and predictable microbial response to dietary fiber supplementation is impeded by the considerable interindividual variability in baseline gut microbiota composition (Mills et al., 2019b). In order for a dietary fiber to generate a specific response, such as an increase in the abundance of a beneficial SCFA-producing species, this bacteria must first be present in the gut microbiota of the individual and must not be susceptible to competitive pressure from other bacteria capable of utilizing the same substrate (Patnode et al., 2019).

Cantu-Jungles *et al.* propose a way to classify dietary fibers based on specificity (Cantu-Jungles et al., 2021). High-specificity fibers are only able to be utilized by a limited number of bacteria, reducing competition and increasing the chance of observing the target response (Cantu-Jungles and Hamaker, 2020). The activity of high-specificity fibers is more predictable and targeted, as it is relatively independent of an individual’s microbiota composition. However, not all individuals may possess the bacteria required for high-specificity fiber utilization and therefore may not be able to respond to supplementation. In contrast, low-specificity fibers can be utilized by a wide variety of gut bacteria (Cantu-Jungles and Hamaker, 2020). The response to low-specificity dietary fiber supplementation is dependent on an individual’s microbiota composition, with highly individualized outcomes, yet the vast majority will demonstrate a response. Multiple factors influence a fiber’s specificity, including chemical complexity, physical properties (Hamaker and Tuncil, 2014), commonality in diet, and involvement in cross-feeding (Cantu-Jungles and Hamaker, 2020). A dietary fiber that requires a wide variety of CAZymes for degradation, such as arabinoxylan (Flint et al., 2012a), or is physically inaccessible due to insolubility, such as *β*-1,3-glucan (Cantu-Jungles et al., 2021) would be considered highly specific, while others that are commonly present in the diet, such as fructo-oligosaccharides, or are extensively involved in cross-feeding, such as resistant starches, may be considered to have low-specificity (Cantu-Jungles et al., 2021). However, most dietary fibers present a mix of high- and low-specificity characteristics, leaving their classification open to interpretation.

While several observational studies have demonstrated an association between a high dietary fiber intake and a lower risk of CRC incidence (Peters et al., 2003; Abebe et al., 2024; Jansen et al., 1999), there exists few prospective studies evaluating the effect of dietary fiber interventions on CRC treatment outcomes. A handful of clinical trials are currently ongoing, looking to examine, respectively, the effect of a mix of inulin, pectin and β-glucan on the response to chemotherapy (NCT07194954), the impact of high-fiber meals on changes in gut microbiota-associated parameters (NCT06349590), and the effect of an inulin-rich vegetable product on the risk of CRC surgical complications (NCT06212817). Given the wide range of dietary fibers utilized in ongoing clinical trials, further research specifically taking into account the impact of dietary fiber type and specificity of microbial fermentation on colorectal surgical healing is needed. Given that colorectal surgical complications are associated with significant morbidity, mortality, and higher rates of cancer recurrence in patients with CRC (Krarup et al., 2014; Mirnezami et al., 2011), the use of personalized dietary fiber supplementation to shift the gut microbiota towards a more anti-inflammatory and pro-regenerative state may be a simple and low-risk option for many patients.

## 5 Dietary fiber and CRC surgical recovery

To this day, surgery remains the main treatment option for both colon and rectal cancers (Abedizadeh et al., 2024; Neumann et al., 2021). CRC surgery involves the resection of the bowel segment harboring the tumor as well as the corresponding mesenteric lymph nodes. In almost all cases, the removal of the affected section of bowel is accompanied by the creation of a surgical anastomosis that reconnects the remaining sections of bowel and ensures intestinal continuity. In a subset of patients ranging from 3 to 23%, this anastomosis fails to heals, allowing intestinal contents to leak out into the abdominal cavity (Rahbari et al., 2010). Anastomotic leak (AL) is a severe surgical complication and is associated with increased mortality, reduced long-term survival, as well as increased local and distant recurrence (Krarup et al., 2014).

Recent work by our team and others demonstrated that the composition of the gut microbiota is causally linked to postoperative recovery and AL. Certain bacterial strains were shown to induce local low-grade inflammation in the bowel, leading in some patients to a pro-inflammatory phenotype and a failure of extracellular matrix restoration within the surgical wound (Hajjar et al., 2023, 2024a). AL was further shown to worsen oncological outcomes and patient quality of life by potentially promoting cancer recurrence and preventing stoma closure (Hajjar et al., 2024b). The permanent use of stoma has been shown to negatively impact patient quality of life, sexual function, and body image (Marinatou et al., 2014). Post-operative complications arising from the poor healing of the surgical anastomosis may have differing and potentially more disruptive impacts on the lives of younger patients with CRC. As this group of patients continues to increase in number, further research is needed to evaluate the specific potential for dietary fiber supplementation as a therapeutic adjuvant for this section of the population.

**Figure 1:**
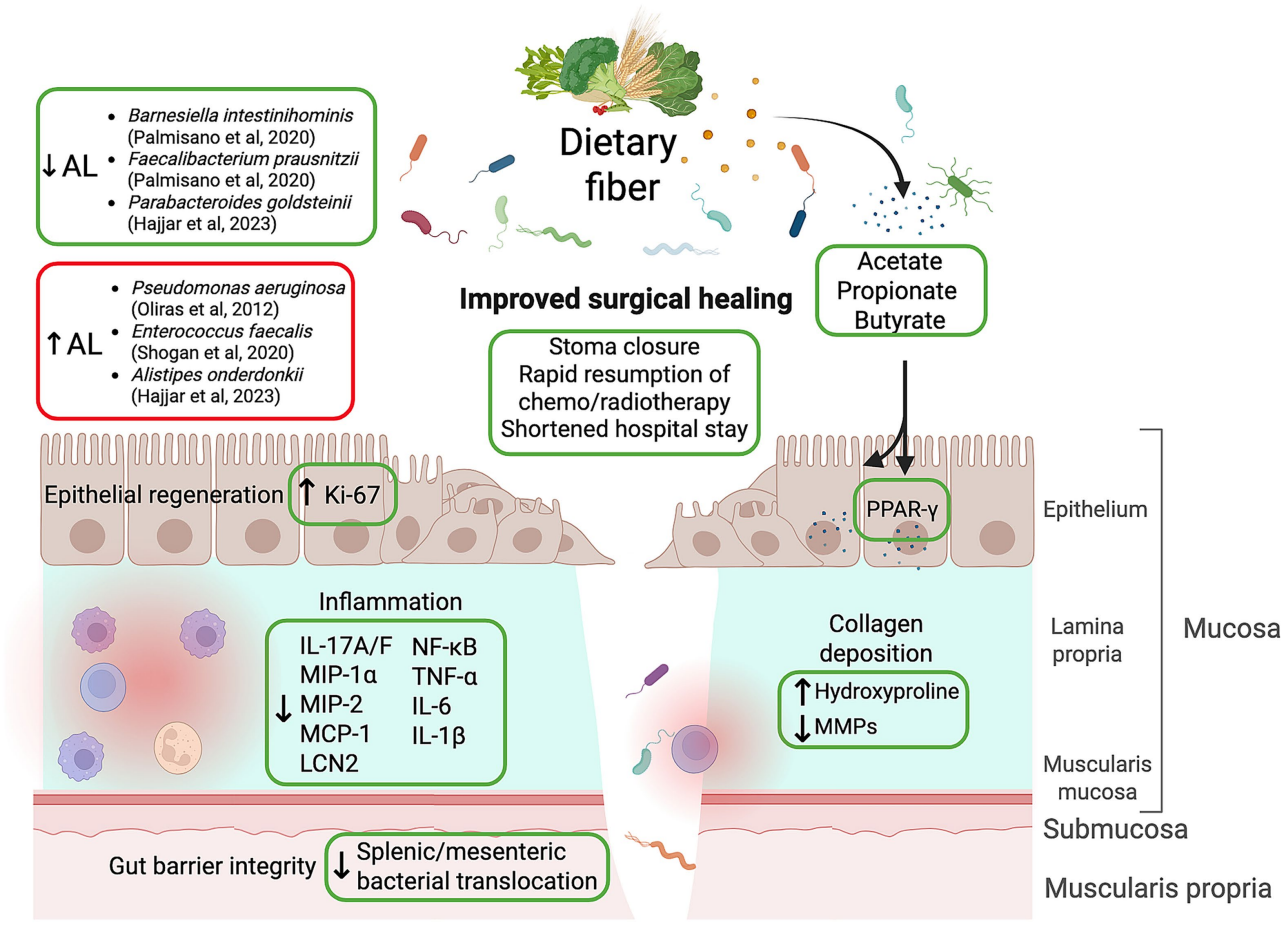
Dietary fiber fermentation by gut microbiota enhances colon surgical recovery. Dietary fibers are fermented by gut bacteria in the colon to produce SCFA, the majority of which are absorbed by colon epithelial cells, with a smaller fraction reaching the submucosa and entering the systemic circulation. SCFA have diverse physiological effects that help to improve intestinal healing following surgery. SCFA promote the proliferation of healthy colon epithelial cells, increase collagen deposition, and upregulate pathways involved in maintaining gut barrier integrity, leading to decreased bacterial translocation and lower immune activation. SCFA also exert anti-inflammatory effects by directly modulating immune cell activation and cytokine production. Enhanced surgical recovery has many benefits for patients, including decreased risk of a permanent stoma, shorter hospitalization, and quicker resumption of adjuvant chemo and radiotherapy. AL, anastomotic leak; PPAR-*γ*, peroxisome proliferator-activated receptor-gamma; MMPs, matrix metalloproteinases; IL-17A/F, interleukin-17 A/F; MIP-1*α*, macrophage inflammatory protein-1 alpha; MIP-2, macrophage inflammatory protein-2; MCP-1, monocyte chemoattractant protein-1; LCN2, lipocalin-2; NF-κB, nuclear factor kappa-light-chain-enhancer of activated B cells; TNF-α, tumor necrosis factor-alpha; IL-1β, interleukin-1 beta; IL-6, interleukin-6. Illustrations were created with BioRender.com.

Our data and that of other teams also showed that a western diet leads to impaired anastomotic healing by promoting collagen-degrading bacteria, highlighting the potential for microbiota-modifying dietary interventions in shaping patient CRC surgical outcomes (Gaines et al., 2020; Hajjar et al., 2021a). As a proof of concept, we showed that a two-week dietary supplementation with inulin or galacto-oligosaccharides (GOS) shaped the preoperative gut microbiota and increased the production of SCFA in a mouse colon anastomosis surgical model, leading to improved healing at the microscopic and macroscopic levels (Marinatou et al., 2014). Quantitatively, mice receiving inulin-supplemented diets possessed a higher percentage of proliferating colonocytes, assessed by the number of Ki-67-positive cells in histological colon samples (Hajjar et al., 2021a) (Figure 1). In addition to increased mucosal continuity, increased collagen deposition, measured by hydroxyproline content and extracellular matrix metalloproteinase (MMP) activity, was also seen in the submucosal layer, which crucially provides a provisional matrix for wound healing (Hajjar et al., 2021a; Bosmans et al., 2015) (Figure 1). In murine models of local and systemic CRC, our team further showed that dietary fiber supplementation, specifically with inulin, increased gut barrier integrity via the production of SCFA, therefore preventing the escape and local implantation of CRC cells as well as alleviating cancer-promoting systemic inflammation and distant liver metastasis (Hajjar et al., 2024b).

Interestingly, microbial modulation of gut barrier integrity and inflammation seems to be associated with the activation of the PPAR-*γ* receptor (Figure 1), potentially paving the way for the use of dietary fibers to stimulate this pathway and improve patient outcomes (Hajjar et al., 2024b,c). These data indicate that dietary fibers could be pivotal in reversing the pro-inflammatory and gut barrier deteriorating effects of certain CRC-associated pathobionts, therefore improving surgical outcomes. However, the direct effect of different dietary fibers on the expansion and pathological activity of specific strains associated with surgical healing requires further investigation.

## 6 Conclusions and perspectives

The gut microbiota has been linked to CRC development by its role as a mediator of diet-associated risk factors and by the pro-carcinogenic properties of certain pathobionts. On the other hand, the gut microbiota plays an important role in intestinal health and CRC prevention through the production of bacterial metabolites, notably SCFA derived from the fermentation of dietary fibers. Moreover, the gut microbiota has been shown to influence patient response to CRC surgical treatment, notably by regulating inflammation and reinforcing the gut barrier.

A limited number of interventional studies have evaluated whether increasing dietary fiber consumption long-term leads to reduced CRC incidence. There exist a large number of confounding dietary risk factors, including the consumption of pro-carcinogenic compounds associated with CRC development, that may limit the preventative potential of dietary fiber supplementation (Mathers et al., 2012). However, dietary interventions of less than a week in duration have been shown to substantially alter microbiota composition and the production of SCFA and bile acids (David et al., 2014), opening the door for short-term dietary fiber supplementations pre- or post-surgery that improve surgical recovery, enhance gut barrier integrity, and reduce chronic low-grade inflammation. A disruption in gut barrier function has been described for several diseases, including CRC and several chronic inflammatory diseases, most notably IBD, as well as multiple systemic diseases including rheumatoid arthritis, Alzheimer’s disease and Parkinson’s disease (Neurath et al., 2025). The pathogenesis of these diseases may be partially attributed to increased local and systemic immune activation and pro-inflammatory cytokine production in response to increased bacterial translocation across the intestinal barrier. This raises the possibility of enhancing clinical responses and reducing gastrointestinal symptoms for patients undergoing neoadjuvant or adjuvant chemotherapy, radiotherapy or immunotherapy through microbiota-modulating dietary fiber interventions aimed at reinforcing the gut barrier.

Previous groups have examined the impact of combining dietary fiber supplementation with chemotherapy *in vivo*, resulting in increased survival (Taper and Roberfroid, 2000) and improved gut barrier function, resulting in reduced treatment-induced enterocolitis and decreased weight loss (Mao et al., 1996). The gut microbiota has been shown to influence chemotherapy efficacy and toxicity through the bacterial metabolism of certain drugs, leading to their inactivation, activation, or transformation into a more toxic form (Spanogiannopoulos et al., 2016). A well know example is the transformation of the CRC chemotherapy drug irinotecan by bacterial *β*-glucuronidase to a form that is toxic to intestinal epithelial cells, leading to exacerbated chemotherapy-induced diarrhea, a side effect observed in up to 80% of patients (Spanogiannopoulos et al., 2016; Stein et al., 2010). Dietary fiber has been shown to reduce β-glucuronidase activity, also associated with the metabolic activation of dietary carcinogens, in a fiber-specific manner *in vivo* (Shiau and Chang, 1983; Freeman, 1986) and in humans (Wu et al., 2011), highlighting the potential for dietary fiber interventions during chemotherapy.

Aside from direct microbiota-mediated effects, SCFA resulting from the fermentation of dietary fibers have immunomodulatory properties that may potentiate the anti-tumor effects of chemotherapy and immunotherapy. Butyrate has been shown to enhance chemotherapy efficacy by promoting CD8 + antitumor immunity in mice (He et al., 2021). Butyrate supplementation specifically improved the efficacy of the chemotherapy agent oxaliplatin through the inhibition of HDAC and increased transcription of genes involved in the Il-12 signaling pathway, resulting in enhanced CD8 + T cell activation and proliferation, and increased anti-tumor immunity (He et al., 2021). Butyrate and propionate have also been shown *in vitro* and *ex vivo* to increase anti-tumor immunity by upregulating IFNγ production and MHC-I expression in CRC cells, leading to increased antigen-presenting capacity and CD8 + T-cell activation (Mowat et al., 2023).

More recently, the immunomodulatory effects of the gut microbiota have been explored in the context of patient response to immune checkpoint inhibitor (ICI) immunotherapy in melanoma (Vétizou et al., 2015; Gopalakrishnan et al., 2018; Routy et al., 2018) and non-small cell lung cancer (Routy et al., 2018). Insufficient dietary fiber intake was also associated with lower survival in melanoma patients undergoing ICI therapy while dietary fiber supplementation in combination with ICIs led to decreased tumor size *in vivo* compared to ICIs alone (Spencer et al., 2021). Another study showed that supplementation with inulin gel increased the efficacy of anti-PD-1 treatment in a CT-26 CRC syngeneic tumor model, driven by an increase in systemic antitumor CD8 + T cells and intratumoral CD8 + and CD4 + T cells specific to the CT-26 epitope AH1 (Han et al., 2021). The authors additionally demonstrated *in vitro* that SCFA contribute to an expansion of memory anti-tumor CD8 + T cells (Han et al., 2021), which is consistent with other studies demonstrating a positive correlation between fecal SCFA levels and patient response to ICIs (Botticelli et al., 2020; Nomura et al., 2020). Crucially, while ICIs have been shown to benefit patients with microsatellite-instability-high (MSI-H) or mismatch-repair-deficient (dMMR) tumors, these represent only 15% of CRCs, while tumors that have proficient mismatch repair (pMMR) or are microsatellite stable (MSS) are less immunogenic and therefore less responsive to immunotherapy (André et al., 2020). However, there exist several emerging therapeutic strategies, including modulation of the gut microbiome, aimed at increasing immunogenicity in the tumor micro-environment (Chen and Zhou, 2025).

Neo-adjuvant radiotherapy is used, either alone or in combination with chemotherapy, in the vast majority of rectal cancer cases (Sebag-Montefiore et al., 2009; Sauer et al., 2004). While radio-ablation can successfully be used to reduce tumor size in more anatomically isolated rectal cases, the localization of colon tumors within the peritoneal cavity is prohibitive due to the damage caused by radiation to surrounding healthy tissues (Neumann et al., 2021). Even with these precautions, radiation-induced toxicity and gastrointestinal dysfunction is common. One study demonstrated that following a high fiber diet during pelvic radiotherapy reduced gastrointestinal toxicity up to one year post-treatment (Wedlake et al., 2017). Another study demonstrated lower serum white blood cell count and reduced mucosal inflammation in rectal tissues of patient undergoing radiotherapy for rectal cancer following a 2 week supplementation with fiber-rich oat bran alone or administered as a synbiotic (Stene et al., 2025). Beyond reducing gastrointestinal symptoms, the modulation of the gut microbiota may improve treatment efficacy, potentially via the production of butyrate, as demonstrated by an increase in radiation-induced cell death in patient-derived CRC organoids treated with low concentrations of butyrate, all while also exhibiting protective effects in healthy tissue organoids (Park et al., 2020).

Finally, while short-term dietary fiber supplementation has the potential to improve treatment efficacy and reduce gastrointestinal symptoms in patients undergoing CRC treatment, significant heterogeneity in individual responses to dietary fiber supplementation has been observed in clinical settings due to baseline differences in microbiota composition (Hoffmann Sardá et al., 2025). Several studies have shown that individuals may be classified as responders or non-responders post-intervention based on changes in microbial or host parameters, depending on the study objectives (Kok et al., 2023). Given these inconsistencies, the identification of biomarkers that predict patient response to a dietary fiber would allow for the pre-stratification and optimization of dietary interventions. Crucially, while the identification of predictive biomarkers of response to several dietary fibers, including an increase in fecal propionate in response to arabinoxylan (Nguyen et al., 2020), an increase in *Bifidobacterium adolescentis* and *Parabacteroides diastonis* in response to resistant starch type 4, an increase in *Ruminococcus bromii* and *Eubacterium rectale* in response to resistant starch type 2 (Martínez et al., 2010), and an increase in *Bifidobacteria* in response to inulin (Kolida et al., 2007), has been shown in healthy volunteers, there exists a lack of studies characterizing microbial and host response to dietary fiber in patients with CRC, who have been shown to harbor distinct microbiome characteristics (Marchesi et al., 2011; Purcell et al., 2017).

In conclusion, recent evidence has shown that short-term dietary fiber supplementation prior to CRC surgical treatment represents a viable option to improve intestinal healing and reduce the risk of postoperative complications, which have been associated with a greater risk of recurrence and poorer long-term survival (Hajjar et al., 2024b). In addition to reinforcing the gut barrier, potentially decreasing low-grade systemic inflammation associated with gut barrier dysfunction (Neurath et al., 2025), microbiota-modifying dietary fiber interventions have also shown important immunomodulatory properties, which may enhance the treatment efficacy of chemotherapy, immunotherapy and radiotherapy administered pre- or post-surgery. Importantly, dietary fiber supplementation may also reduce gastrointestinal symptoms associated with cancer therapies, improving patient quality of life and increasing treatment adherence. Finally, there exist considerable opportunities for the advancement of precision medicine in the field of microbiota-modifying dietary interventions, allowing for the pre-stratification of patients and the optimizing of intervention outcomes.
